# Effective Inhibition of Invasive Pulmonary Aspergillosis by Silver Nanoparticles Biosynthesized with *Artemisia sieberi* Leaf Extract

**DOI:** 10.3390/nano12010051

**Published:** 2021-12-25

**Authors:** Enas M. Ali, Basem M. Abdallah

**Affiliations:** 1 Department of Biological Sciences, College of Science, King Faisal University, Al-Ahsa 31982, Saudi Arabia; eabdelkader@kfu.edu.sa; 2 Department of Botany and Microbiology, Faculty of Science, Cairo University, Cairo 12613, Egypt

**Keywords:** *Aspergillus fumigatus*, antifungal, invasive pulmonary aspergillosis, calotropis gigantea

## Abstract

*Aspergillus fumigatus* is one of the most common fungal pathogens that can cause a diversity of diseases ranging from invasive pulmonary aspergillosis (IPA) and aspergilloma to allergic syndromes. In this study, we investigated the antifungal effect of silver nanoparticles biosynthesized with *Artemisia sieberi* leaf extract (AS-AgNPs) against *A. fumigatus* in vitro and in vivo. The biosynthesized AS-AgNPs were characterized by imaging (transmission electron microscopy (TEM)), UV−VIS spectroscopy, X-ray diffraction (XRD), and Fourier transform infrared spectroscopy (FTIR). The microdilution method showed the antifungal activity of AS-AgNPs against *A. fumigatus*, with an MIC of 128 µg/mL. AS-AgNPs significantly inhibited the growth of hyphae in all directions, as imaged by SEM. Additionally, TEM on biofilm revealed invaginations of the cell membrane, a change in the vacuolar system, and the presence of multilamellar bodies within vacuoles. Interestingly, AS-AgNPs displayed low cytotoxicity on the A549 human lung cell line in vitro. Treatment of an invasive pulmonary aspergillosis (IPA) mouse model with AS-AgNPs demonstrated the potency of AS-AgNPs to significantly reduce lung tissue damage and to suppress the elevated levels of pro-inflammatory cytokines, tumor necrosis factor-alpha (TNF-α), interleukin-1 (IL-1), and interleukin-17 (IL-17). The therapeutic potential of AS-AgNPs was found to be due to their direct action to suppress the fungal burden and gliotoxin production in the lungs. In addition, AS-AgNPs reduced the oxidative stress in the lungs by increasing the enzymatic activities of catalase (CAT) and superoxide dismutase (SOD). Thus, our data indicate the biosynthesized AS-AgNPs as a novel antifungal alternative treatment against aspergillosis.

## 1. Introduction

*A. fumigatus* is the major pathogenic agent responsible for approximately 90% of IPA cases and is considered one of the most significant invasive fungal infections of worldwide concern [1,2]. The risk of developing infections increases in patients with underlying debilitating diseases, such as cancer, chronic lung diseases, transplantation, or immune system impairment [3,4].

In chronic forms of IPA, *A. fumigatus* develops a thick biofilm that promotes its persistence [5]. Biofilm formation by *A. fumigatus* is reported as one of the most important virulence factors in IPA [6]. In addition, several secondary metabolites secreted by *A. fumigatus* are required for infection [7], including gliotoxin (GT), a secondary metabolite that belongs to the epipolythiodioxopiperazine class of mycotoxins [8] and is found in the sera of patients with IPA [9]. GT inhibits the host immune response and is used as a potential virulence factor in IPA infection [10,11].

IPA is associated with a high risk of unsuccessful treatment and mortality. Due to the common eukaryotic cell structure of fungi and humans, a limited number of antifungal drugs are tolerated by the host and available for therapeutic purposes [12]. Triazoles are the first-line antifungal drugs used in the treatment of IPA [13]. However, there is increasing evidence of triazole resistance in clinical populations of *A. fumigatus* [14]. Amphotericin B (AMB) is the current standard medication for IPA, although it has major side effects, such as electrolyte imbalance and progressive renal failure [15]. In addition, approximately only 33–54% of patients with IPA respond to AMB therapy due to antibiotic resistance [16].

Thus, there is a crucial need for the expansion of novel therapeutic approaches [17]. In this context, natural antifungal drugs including plant extracts and essential oils are used as anti-*Aspergillus* drugs against *A. fumigatus* [18,19]. Engineered, biofabricated silver nanoparticles (AgNPs) have been widely used in many biomedical applications due to their antimicrobial properties [20,21] and AgNPs were effectively used to combat *A. fumigatus* [22,23,24]. We recently reported the efficient inhibition of candidiasis by biosynthesized AgNPs using the plant extract of *Calotropis gigantea* or *Lotus lalambenesis* [25,26].

*A. sieberi* (Compositae family) is a well-known medicinal plant in Middle East traditional medicine. The flowering parts used for the treatment of gangrenous ulcers, infectious ulcers, and inflammations. It is used as a carminative, relieves inflammation and abscesses, and prevents leprosy [27]. It is also used for the skin as an antimicrobial [28], an insecticidal [29], a nematocidal [30], an anti-malaria agent [31], and an anti-coccidiosis agent [32]. In this study, we investigated the antifungal effect of biosynthesized AgNPs using the leaf extract of *A. sieberi* on *A. fumigatus* in vitro and in vivo in an IPA mouse model. Our data demonstrated the inhibitory effect of AS-AgNPs on fungal growth and biofilm formation in vitro and gliotoxin production, lung oxidative stress, and the production of pro-inflammatory cytokines in vivo.

## 2. Materials and Methods

### 2.1. Microorganism and Culture Conditions

A strain of *A. fumigatus* was previously isolated by our group from an immunocompromised patient with IPA used in this study [33]. *A. fumigatus* was cultured on peptone yeast extract glucose (PYG: peptone 1 g; yeast extract 1 g; glucose 3 g; per liter of distilled water) agar slants at room temperature. For the preparation of conidial suspension, A. fumigatus was grown on PYG for 6 days at 35 °C and the conidia were harvested, as described previously [34].

### 2.2. Plant Material

Leaves of *Artemisia sieberi* were collected from Eastern Province, Al-Hassa, Saudi Arabia. The sample was identified taxonomically and authenticated by the Department of Botany and Microbiology, Cairo University, where a voucher specimen was deposited (voucher no. M8E7). Next, 50 g of leaf powder was added to 500 mL of distilled water, and the mixture was heated at 80 °C for 4 h with stirring. The resulting extract was stored at 4 °C for further use.

### 2.3. Biosynthesis and Characterization of AgNPs

AgNPs were synthesized, purified, and characterized, as discussed earlier [25]. Briefly, 10 mL of 1 mM AgNO_3_ was added to 50 mL of plant extract to be converted into AgNPs at room temperature in the dark, and the mixture was heated at 80 °C for 3 h with stirring. Complete reduction was confirmed by a change in color from colorless to brown. The biosynthesized AgNPs were separated by centrifugation. The final green-synthesized AgNPs were signified as AS-AgNPs, freeze-dried, and stored at 4 °C for further experiments.

The UV–VIS absorption spectrum of biosynthesized AgNPs was carried out by a PerkinElmer Lambda 35 double-beam spectrophotometer. For TEM analysis, the purified AgNPs were examined by transmission electron microscopy (TEM, H7100; Hitachi Ltd., Tokyo, Japan). The X-ray diffraction (XRD) (Unisantis XMD-300, Geneva, Switzerland) measurement of AgNPs was done using a Cu-Kα radiation source in a wide range of Bragg angles 2θ at a scanning rate of 0.388/min in a powder diffractometer (PANalyticalXper PRO model X-ray diffractometer) at a voltage of 50 kV and a current of 30 mA. For Fourier transform infrared spectroscopy (FTIR) (Thermo Nicolet AVATAR 370, Waltham, MA, USA) analysis, 100 mL of residual solution formed after the synthesis of AgNPs was centrifuged at 10,000 rpm for 15 min. Then, the suspension was dissolved in 10 mL of sterile distilled water. The purified suspension was frizzed to obtain the dried powder. Then, the dried AgNPs were analyzed through an FTIR spectrophotometer.

### 2.4. In Vitro Antifungal Susceptibility Testing

The minimum inhibitory concentration (MIC) of AS-AgNPs were determined according to Clinical and Laboratory Standards Institute Document M38-A2 [35]. AgNPs were used over concentration ranges of 1–512 μg/mL. *A. fumigatus* spores were harvested from stock cultures and their concentration adjusted to 1.0 × 10^6^ CFU. Then, the spores were put on the center of PDA plates containing different concentrations of AgNPs (512, 256, 128, 64, 16, and 4 μg/mL). The plates were incubated at 35 °C, and the growth of *A. fumigatus* was observed after 7 days. The MIC was identified as the lowest concentration of the AgNPs that did not show any growth of fungal colonies on the agar plates [36].

### 2.5. In Vitro Antifungal Activity Assay

Antifungal activity was assessed by the agar well diffusion method [37]. The nutrient agar plates were swabbed with a 24 h broth culture of A. fumigatus and kept for 15 min in a laminar chamber. Wells were made in agar plates using a sterile cork borer of 5 mm. AS-AgNPs and amphotericin B at various concentrations were prepared, and 20 µL of each concentration was added to each well. DMSO was used as the negative control. The plates were incubated at 37 °C for 24 h. The diameters of the zones of inhibition were measured by using the antibiotic zone measuring scale.

### 2.6. Time Kill Assay

Briefly, 10 mL of *A. fumigatus* conidial suspension (1 × 10^6^ conidia/mL) was added to 10 mL of RPMI-1640 medium alone (negative control) and to amphotericin B (positive control) or AS-AgNPs diluted in 10 mL of RPMI-1640 medium. Cultures were agitated at 37 °C. At different time intervals (0, 4, 8,12, 16, and 24 h), a sample of 0.1 mL was removed from each test suspension and spread on PDA plates and then incubated at 37 °C for 48 h. The time–kill curves were made by plotting the colony-forming units (CFU) per milliliter surviving at each time interval in the presence of various antimicrobial drugs [18].

### 2.7. Scanning Electron Microscopy (SEM)

*A. fumigatus* was treated for 12 h with AS-AgNPs in RPMI-1640 medium. For SEM analysis, fungal hyphae were fixed in 0.25% glutaraldehyde and 1% osmic acid (Sigma-Aldrich, Burlington, MA, USA) and dehydrated by a series of increasing concentrations of ethanol. After replacement of ethanol with acetone, they were dried using carbon dioxide. The fungal cells were coated with gold palladium and examined using a JEOL field emission scanning electron microscope (model ISM 6400 F) at 12–15 kV [38].

### 2.8. Effect of AS-AgNP Treatment on A. fumigatus Biofilm Morphology and Structure

Fungal biofilms were prepared on 96-well flat-bottomed polystyrene plates by using conidia harvested from the aerial static culture according to the method described by [39]. *A. fumigatus* conidia were suspended in RPMI-1640 medium. For ultrastructural examination, *A. fumigatus* biofilms were fixed with 2.5% glutaraldehyde in RPMI-1640 medium for 4 h at room temperature and rinsed with distilled water. Post-fixation was carried out in 1% OsO_4_ in H_2_O for 2 h. The specimens were fixed in 2% agar in H_2_O and then were dehydrated and embedded in epoxy resin. Ultrathin sections were stained with uranyl and examined under a Zeiss EM900 electron microscope (Carl Zeiss Microscopy Deutschland GmbH, Oberkochen, Germany) operated at 80 kV equipped with a 30 μm objective aperture [40].

### 2.9. Cell Culture and Cytotoxicity Assay

Mouse bone-marrow-derived mesenchymal stem cells (BMSCs) were isolated and cultured from C57BL/6J mice according to our previously described protocols [41]. Briefly, cells were flushed out from mouse bone, suspended in PBS, filtered through a 70 μm filter, and cultured in RPMI-1640 medium supplemented with 12% FBS (Gibco Invitrogen, Waltham, MA, USA) and 1% penicillin/streptomycin (P/S) (Gibco Invitrogen, Waltham, MA, USA). After 24 h of culture at 37 °C, non-adherent cells were collected by centrifugation and re-cultured in fresh medium. The A549 human lung carcinoma epithelial-like cell line was obtained from the American Type Culture Collection, ATCC (#CCL-185). Cells were cultured in DMEM (Dulbecco’s Modified Eagle Medium) supplemented with 1% penicillin/streptomycin (P/S; Gibco Invitrogen, Waltham, MA, USA) and 10% fetal bovine serum heat inactivated (FBS; Gibco Invitrogen, Waltham, MA, USA).

For cytotoxicity assay, the MTT (3-(4,5-dimethylthiazol-2-yl)-2,5-diphenyltetrazolium bromide) cell proliferation assay kit (Sigma-Aldrich, Burlington, MA, USA) was used to measure the cell viability according to the manufacturer’s instruction kit. Cells were treated with either amphotericin B or AS-AgNPs at different concentrations in 96-well plates for 48 h. Cells were then incubated with a medium containing 0.5 mg/mL of MTT to metabolize to formazan. The optical density was measured at 550 nm using an ELISA plate reader [42]. Values were represented as a percentage of control, non-treated cells.

### 2.10. In Vivo Experiment Design

Swiss albino mice (10 weeks old) were obtained from the animal house, National Research Center. The animals were housed in a controlled temperature of 25 ± 2 °C in a 12 h dark/light cycle with standard diet and water ad libitum. The procedure of the in vivo IPA experiment was approved by the Faculty of Science Institutional Animal Care and Use Committee (IACUC), Cairo University, Egypt (CUFS/F/10/13).

Twenty-four male mice were grouped into 3 groups (*n* = 8). Neutropenia was induced in all mice by a single intraperitoneal (ip) administration of cyclophosphamide (150 mg/kg), as described previously [43]. The first group of mice was assigned as control mice that were injected intraperitoneally with Hank’s Balanced Salt Solution (HBSS). To induce IPA in the mice, the rest of the neutropenic mice were infected with conidia of *A. fumigatus* (administered by intranasal instillation), 3 days post-neutropenic induction, as described previously [44]. Then, 24 h after fungal inoculation, the IPA mice were intratracheally instilled either with HBSS (IPA control group, second group) or with AS-AgNPs (0.5 μg/g, third group) [45]. The mice were examined at 3 days post-instillation.

### 2.11. Histological Study

The lung was fixed in 10% buffered formalin and then embedded in paraffin, sectioned, and stained with hematoxylin and eosin (H&E) and periodic acid Schiff–stained (PAS). Tissue sections were imaged using a Nikon 80i light microscope (Nikon Corporation, Tokyo, Japan).

### 2.12. Biochemical Assays

Blood samples were collected from the dorsal aorta of mice, and sera were separated by centrifugation. Serum biochemical markers, including aspartate aminotransferase (AST), alanine aminotransferase (ALT), urea, and creatinine, were determined according to the instruction manual of commercial available kits from Abcam (Cambridge, UK).

Lung tissues were homogenized in HBSS, incubated in an ice bath, and centrifuged at 12,000 rpm for 15 min at 4 °C. The supernatants were collected for measurements. CAT activity was determined by measuring the decrease in absorbance of hydrogen peroxide at 240 nm following the method of [46]. SOD activity was determined using the adrenochrome test, which relies on the ability of SOD to inhibit the autoxidation of epinephrine in an alkaline medium according to the method of [47], and the MDA level was measured by the thiobarbituric acid test [48]. TNF-α, MPO, IL-1, and IL-17 were estimated by the ELISA kit (MyBioSource, Inc., San Diego, CA, USA) according to the manual instructions.

### 2.13. Determination of Fungal Load in Lung Tissue

For tissue burdens, aliquots of tissue were cultured on Potato dextrose agar, PDA agar plates using serial 10-fold colony count dilutions. Plates were incubated at 37 °C until colonies could be counted. The undiluted homogenate was saved at 4 °C until initial cultures were counted. When the undiluted homogenate had no counts, the entire organ homogenate was cultured. Counts were expressed as CFUs [44].

### 2.14. Assessment of Gliotoxin in Lung Tissue

Gliotoxin was extracted from infected lung tissues according to the method of Richarad and DeBey (1995). Briefly, lung samples were soaked in plastic bags with a mallet, mixed with 5 mL of distilled water, and then transferred to a tissue homogenizer. The samples were homogenized, and then 10 mL of HCl was added to the homogenates on a shaker for 30 min. The mixtures were extracted with 200 mL of chloroform, passed through 5 g of sodium sulfate, and then filtered through a glass filter. The eluates were dried on Rotavap at 30 °C. Gliotoxin was eluted with 15 mL of ether:acetone (95:5, *v*/*v*) in a 25 mL beaker and evaporated under a stream of nitrogen at 30 °C. Samples were then quantitatively transferred to vials using ether:acetone (95:5, *v*/*v*), and the solutions in the vials were dried. 

### 2.15. Statistical Analysis

All values are expressed as the mean ± standard deviation (SD) of at least 3 independent experiments. Power calculation was performed for 2 samples using unpaired Student’s *t*-test (2-tailed) assuming equal variation in the 2 groups. Differences were considered statistically significant at * *p* < 0.05 and ** *p* < 0.005.

## 3. Results

### 3.1. Biosynthesis and Characterization of AS-AgNPs

**Figure 1:**
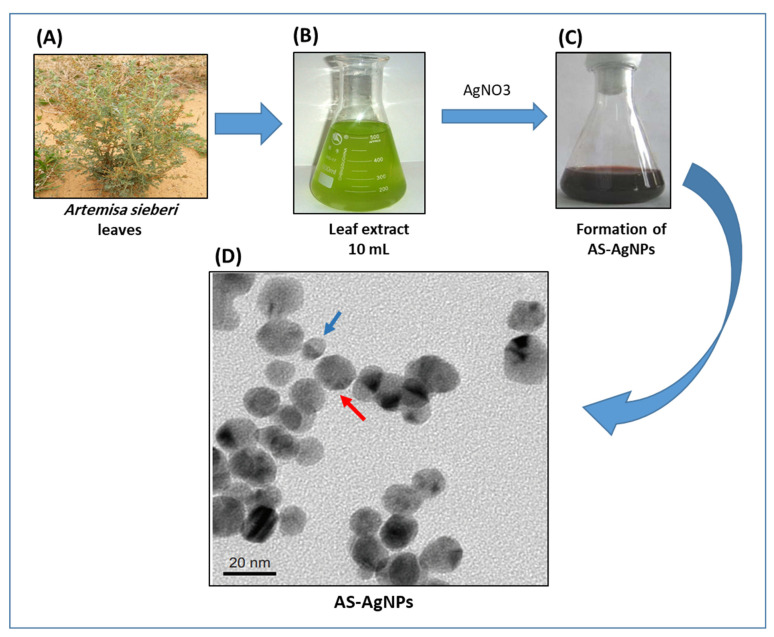
Biosynthesis of AS-AgNPs using *A. sieberi* leaf extract. (**A**–**C**) The biosynthesis of AS-AgNPs was performed by treating the leaf extract of *A. sieberi* with 1 mM AgNO_3_ solution at 27 °C under dark conditions. (**D**) TEM micrograph of AS-AgNPs synthesized from *A. sieberi* leaf extract. TEM shows the size and shape of monodisperse AgNPs as a diameter range of 8 (blue arrow) to 22 (red arrow) nm and spherical.

**Figure 2:**
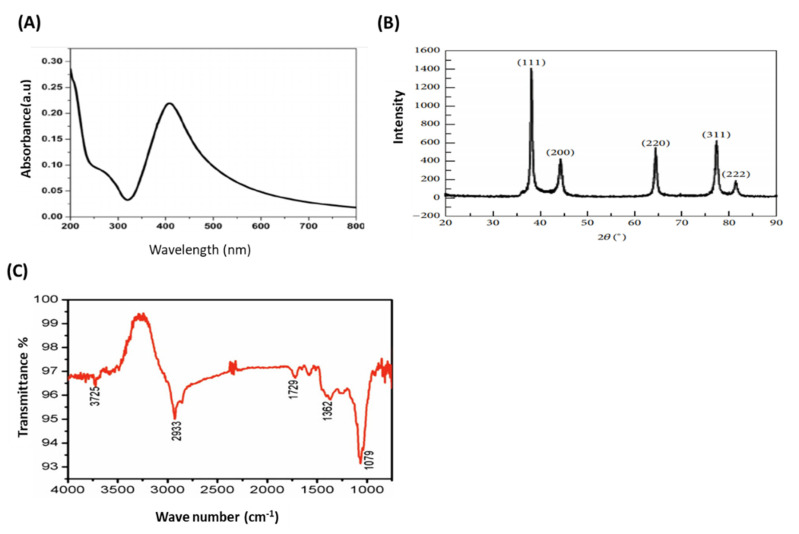
Confirmation of biosynthesized AS-AgNPs. (**A**) UV–VIS spectrum of AS-AgNPs. The highest absorbance peak was at about 450 nm, corresponding to the plasmon resonance of AS-AgNPs. (**B**) The XRD spectrum recorded for AS-AgNPs showed four distinct diffraction peaks 38.25°, 46.37°, 64.60°, and 77.62° corresponding to 1 1 1, 2 0 0, 2 2 0, and 3 1 1 planes for silver, respectively. (**C**) FTIR spectrum of AS-AgNPs. The peak at 3725 cm^−1^ confirms strong, broad O–H stretches of carboxylic bands. The peak at 1079 cm^−1^ demonstrates carbonyl stretching bands. The peak at 1362 cm^−1^ specifies alkene groups. The peaks at 1729 cm^−1^ and 2933 cm^−1^ specify the presence of alkane groups.

AS-AgNPs were biofabricated from an aqueous leaf extract of *A. sieberi*. The change in the color of the extract of *A. sieberi* after addition of silver nitrate from colorless to dark brown was identified (Figure 1A–C). This brown color revealed the formation of AgNPs. The biosynthesis of AS-AgNPs was confirmed by transmission electron microscopy, which showed that the purified AS-AgNPs were spherical in shape with a diameter range of 8–22 nm (Figure 1D). The results of the UV–VIS spectrum showed a Surface plasmon resonance, SPR peak at 407 nm, which is a characteristic peak of AgNPs (Figure 2A). The XRD pattern of the biosynthesized AS-AgNPs corresponded to that of AgNPs. The XRD results showed four strong peaks in the whole spectrum of 2θ values ranging from 30 to 80. The analysis of the XRD spectrum confirmed that the biosynthesized AS-AgNPs were in the form of nanocrystals, obvious by the peaks at 2θ values of 38.25°, 46.37°, 64.60°, and 77.62°, which correspond to 1 1 1, 2 0 0, 2 2 0, and 3 1 1 planes for silver, respectively (Figure 2B). The results of FTIR indicated the presence of carboxylic, hydroxyl, alkanes, and carbonyl groups on the surface of biosynthesized AS-AgNPs. The peak at 3725 cm^−1^ indicated strong, broad O–H stretches of carboxylic bands. The peak at 1079 cm^−1^ displayed carbonyl stretching bands. The peak at 1362^−1^ might be due to alkene groups. The peaks at 1729 cm^−1^ and 2933 cm^−1^ might be due to the presence of alkane groups (Figure 2C).

### 3.2. In Vitro Antifungal Activity of AS-AgNPs against A. fumigatus

**Figure 3:**
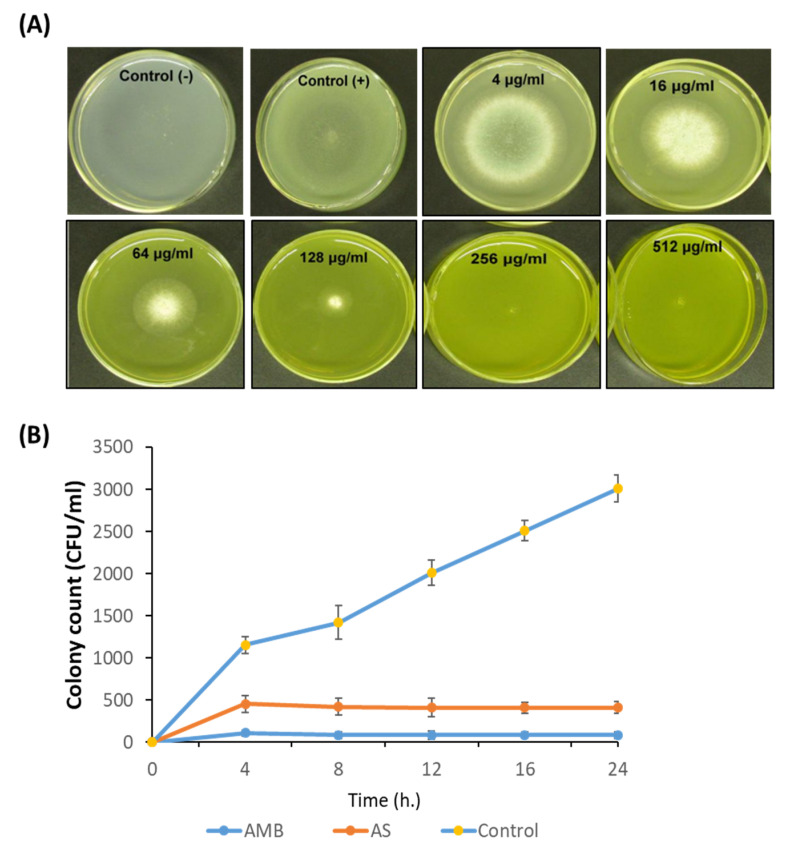
Antimicrobial potential of AS-AgNPs. (**A**) *A. fumigatus* spores were added to the center of PDA plates containing different concentrations of AS-AgNPs (4, 16, 64, 128, 256, and 512 μg/mL). The positive control (amphotericin B) was treated with DMSO (less than 1%), and the negative control was only PDA without fungus. The MIC was identified as the lowest concentration of AS-AgNPs that does not show any growth of fungal colonies on the agar plates. At concentrations of more than 128 μg/mL, there were no colonies visible on the plates. (**B**) Time–kill curves of *A. fumigatus* following exposure to AS-AgNPs and amphotericin B. Values are expressed as means ± SD.

We investigated the anti-*Aspergillus* potential of AS-AgNPs against *A. fumigatus* using the CLSI broth micro-dilution method. After 5 days of incubation, the results displayed that with increasing concentrations of AS-AgNPs, the growth of *A. fumigatus* was significantly repressed. At concentrations of more than 128 μg/mL, no fungal colonies were visible on plates (Figure 3A); therefore, we concluded that the MIC was 128 μg/mL. AS-AgNPs completely inhibited the growth of *A. fumigatus* (Figure 3B). Additionally, we compared the anti-*Aspergillus* potential of different concentrations of AS-AgNPs and amphotericin B using the disk diffusion method. AMB showed a higher antifungal action with an inhibition zone diameter of 35 mm, while AS-AgNPs showed moderate antifungal potential with an inhibition zone diameter of 30 mm (Supplementary Table S1). The MIC values of AMB and AS-AgNPs were 64 and 128 µg/mL, respectively (Supplementary Table S1). Additionally, the time–kill curves showed the fungistatic action of both AMB and AS-AgNPs at 64 µg/mL and 128 µg/mL, respectively, on the growth of *Aspergillus* cells (Figure 3B). After only 8 h of incubation, AS-AgNPs completely inhibited the growth of *A. fumigatus* to zero colonies (Figure 3B). 

### 3.3. Ultrastructural Analysis of the Interaction between AS-AgNPs and A. fumigatus Cells Using Scanning Electron Microscopy

**Figure 4:**
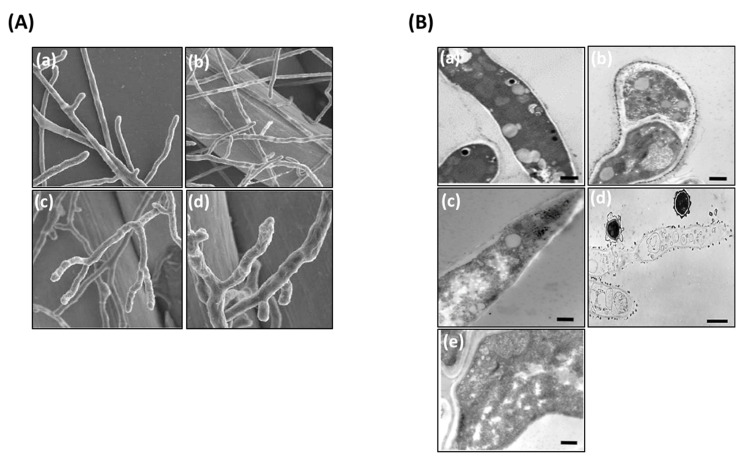
Electron microscopy photographs of *A. fumigatus* after treatment with AS-AgNPs. (**A**) Scanning electron micrographs of *A. fumigatus* treated with (**a**,**b**) saline and (**c**,**d**) AS-AgNPs (128 μg/mL). (**B**) Transmission electron microscopy of *A. fumigatus* biofilm treated with (**a**,**b**) saline and (**c**–**e**) AS-AgNPs (12,000× original magnification, bar = 600 nm).

The effect of AS-AgNPs (128 μg/mL) on *A. fumigatus* hypha was studied using SEM. Untreated filaments displayed linear hyphae, and each new hypha was perpendicular to the main hypha (Figure 4A(a,b)). In contrast, hyphae treated with AS-AgNPs were tortuous and could grow in all directions (Figure 4A(c,d)). Furthermore, AS-AgNP-treated cells were incapable of the production of new secondary hyphae, as displayed by the presence of numerous buds at the apical zone of the hyphae.

### 3.4. Effect of AS-AgNPs on Morphology and Structure of A. fumigatus Biofilm

TEM evaluation showed an advanced loss of cellular structures at cell membrane and cytoplasmic levels (Figure 4B). The fungal biofilms displayed some modifications of the inner membrane, which became irregular in thickness and density; however, internal organelles, such as mitochondria, were well maintained (Figure 4B(b)). In contrast, in AS-AgNP-treated biofilms, the hyphae showed a collapsed cell wall (Figure 4B(c)). The cytoplasm was obviously degenerated because of high vacuolization and no organelles identified. In addition, a large part of the fungal structure was destroyed and disrupted, and many cells appeared empty (Figure 4B(d)). Several cells were damaged, displaying cell wall detachment from the plasma membrane, invaginations of the plasma membrane, and the occurrence of multilamellar structures. The internal structure was less definite and showed higher density (Figure 4B(e)). 

### 3.5. AS-AgNPs Exert No Cytotoxicity on Mouse and Human Cells

**Figure 5:**
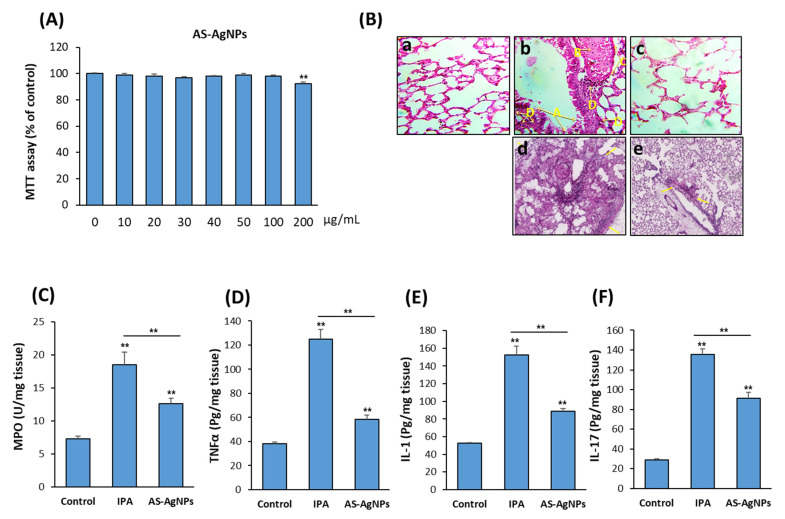
Cytotoxicity of AS-AgNPs on the human lung cancer cell line, A549. (**A**) The dose-dependent effect of AS-AgNPs on the cell viability of human A549 cells was measured by MTT assay after 48 h of treatment. Values are shown as the mean ± SD of three independent experiments. (**B**) Histological sections of lung tissue stained with (**a**–**c**) H&E and (**d**,**e**) periodic acid Shiff (PAS) from 3-day post-AS-AgNP instillation. (**a**) Control mice (HBSS), (**b**,**d**) IPA mice, and (**c**,**e**) IPA mice treated with AS-AgNPs. Arrows in (**b**) specify the following: A; degeneration and desquamation of the lining of the respiratory epithelium of the bronchiole; B, dilatation and congestion of blood vessels; C, thickening in the wall of blood vessels; and D, lymphocytic infiltration in tissue. Arrows in (**d**) and (**e**) specify the boundaries of fungal lesions. Measurements of inflammatory cytokines (**C**) MPO, (**D**) TNF-α, (**E**) IL-1, and (**F**) IL-17 at 3 days post-instillation of IPA mice with HBSS or AS-AgNPs. Data are expressed as the means ± SD (*n* = 8 mice/group). ** *p* < 0.005 compared to control, non-treated cells.

To study the possible use of AS-AgNPs for the treatment of IPA in vivo, we first examined the cytotoxicity of AS-AgNPs on primary mBMSCs and the A549 human lung cancer cell line using cell viability MTT assay. As shown in Figure 5A and Figure S1 AS-AgNPs were not toxic to both mBMSCs and human A549 cells up to a concentration of 100 µg/mL and started to show significant reduction in cell viability at a concentration of 200 µg/mL (Figure 5A). These data demonstrated that AS-AgNPs are safe to be used in vivo in the IPA mouse model.

### 3.6. AS-AgNPs Significantly Reduce Inflammation and Repair Lung Tissue Damage in IPA Mice

The H&E-stained lung sections of control neutropenic mice displayed healthy lung tissues, while IPA mice displayed the incidence of degeneration, desquamation of the lining of the respiratory epithelium of the trachea, expansion and thickening of the alveoli walls, congestion and hemolysis of blood vessels, and lymphocytic cell infiltration (Figure 5B(a–c)). The results of sections stained with PAS showed large fungal lesions described by extensive fungal growth and tissue damage, as revealed by staining (Figure 5B(d,e)). Interestingly, AS-AgNPs did not demonstrate any tissue damage in other organs, including the liver, kidney, heart, and spleen, as shown by histological analysis (Figure S2A). In addition, AS-AgNPs exerted no effects on liver and kidney functions, as revealed by biochemical analysis of serum markers for liver function (AST and ALT) and kidney function (urea and creatinine) (Figure S2B,C).

The lung tissue damage in IPA mice was associated with a significant increase in the production of pro-inflammatory cytokines, including myeloperoxidase (MPO), TNFα, IL-1, and IL-17 (Figure 5C–F). Interestingly, IPA-induced lung tissue damage was significantly repaired in IPA mice treated with AS-AgNPs, as revealed by a significant reduction in fungal abscesses and the normal lung tissue architecture. These histological data were supported by a significant reduction in the levels of MPO, TNFα, IL-1, and IL-17 in AS-Ag-NP-treated IPA mice as compared to control IPA mice (Figure 5C–F).

### 3.7. AS-AgNPs Decrease Colonization of A. fumigatus and Inhibit Gliotoxin Production in Lung Tissue of IPA Mice

**Figure 6:**
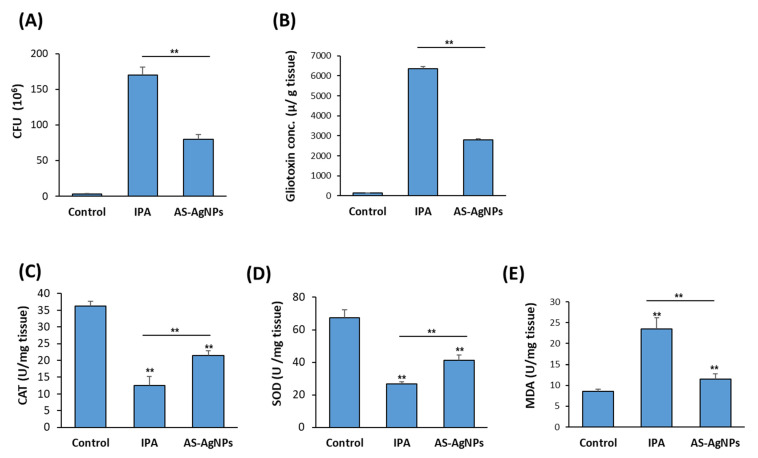
Effect of AS-AgNPs on lung fungal load, gliotoxin production, oxidative stress, and inflammation in IPA mice. Effect of AS-AgNPs on fungal load in lung homogenate of IPA mice (**A**). (**B**) Lung gliotoxin concentration in IPA mice treated with AS-AgNPs. The effect AS-AgNPs on the production of antioxidant enzymes (**C**) CAT, (**D**) SOD, and (**E**) MDA in the lung of IPA mice at 3 days post-instillation. Values are expressed as means ± SD (*n* = 8 mice/group). ** *p* < 0.005 compared to control, non-treated mice.

The mean burden of fungal cells in the autopsied lungs (CFU/g lung tissue) was measured in IPA-treated mice versus control, non-treated IPA mice. The mean burden of fungal cells in the lung was 170 × 10^6^ and 80 × 10^6^ in the IPA control group and the AS-AgNPs group, respectively (Figure 6A). The measurements of gliotoxin production by *A. fumigatus* in IPA mice revealed the efficiency of AS-AgNP treatment in inhibiting gliotoxin production by 56% in IPA mice (Figure 6B).

### 3.8. AS-AgNPs Reduce the Oxidative Stress in IPA Mice

The antioxidant enzyme activities of CAT and SOD (Figure 6C,D) in the lung of non-treated IPA mice significantly decreased, while the levels of MDA significantly elevated compared with the control neutropenic mice group (Figure 6E). Treatment of IPA mice with AS-AgNPs efficiently increased the levels of CAT and SOD but reduced the levels of MDA in IPA mice as compared to the control mice group (Figure 6C–E).

## 4. Discussion

The green synthesis of AgNPs using plant extracts with defined size and morphology is a simple, nontoxic, and eco-friendly method. This is the first study to use *A. sieberi* in the biosynthesis of AgNPs with high antifungal potency against *A. fumigatus* in vitro and in vivo.

The surface plasmon resonance (SPR) spectrum of the synthesized AS-AgNPs from the leaf extract of *A. sieberi* was displayed at 407 nm, which verified the biosynthesis of AgNPs in the reaction medium, as reported by Sathishkumar et al. (2009), who synthesized AgNPs from *Z. zerumbet* tubers, which showed an SRP spectrum at 407–410 nm [49]. This band appears due to the surface plasmon oscillation modes of conduction electrons, which are coupled through the surface to external electromagnetic fields [50]. Our green-synthesized AgNPs with sizes in the range of 8–22 nm were uniform, as revealed by TEM analysis [51], and the FTIR spectrum demonstrated the presence of O–H stretches of carboxylic bands and carbonyl stretching bands, as described previously [52]. Our results might indicate the involvement of the OH functional group in the reduction of Ag+ ions [53]. In this context, OH and carbonyl stretches might be attributed to phytochemicals, such as alkaloids, terpenoids, flavonoids, and phenolics, which are abundantly present in the plant extract [54]. Based on this evidence, it can be inferred that both hydroxyl and carboxyl groups are responsible for the stabilization of silver nanoparticles [55].

AgNPs biosynthesized by different methods and species have been reported to display antifungal potential against *Aspergillus* sp. at different MIC values based on their size, shape, and surface modification [56,57]. Biosynthesized AgNPs using *Cassia roxburghii* leaf extract display maximum inhibitory activity against *A. fumigatus*, with an MIC of 100 μg/mL [58], while biosynthesized AgNPs using *Aloe vera* leaf extracts show antifungal activity with an MIC of 21.8 ng/mL [59]. In addition, AgNPs biosynthesized by *Momordica charantia* and *Psidium guajava* leaf extracts exhibit effective inhibitory action against *A. niger* and *A. flavus* at an MIC of 40 μg/mL [60].

Our results identified the MIC of As-AgNPs against *A. fumigatus* to be 128 μg/mL with moderate antifungal activity as compared to AMB. Although AMB displayed higher antifungal potential, it interacts with mammalian sterols, such as cholesterol, which can lead to harmful effects [61]. Additionally, several studies have shown that only 50% of patients with invasive mold aspergillosis infections display a favorable response to AMB treatment and the survival rate at 12 weeks was only 59%. Higher rates of nephrotoxicity and hypokalemia were seen. Therefore, there appears to be no benefit, and significant harm, with a higher AMB dosing strategy in the treatment of invasive aspergillosis [62]. Furthermore, AMB therapy has major side effects, such as fever and shivering. Typical side effects also include electrolyte imbalance and progressive renal failure. Approximately 33–54% of patients with IPA respond to AMB therapy [16]; however, mortality exceeds 64–90%, despite adequate treatment [63]. Therefore, AgNPs have been used due to their intrinsic antifungal activity or as a drug delivery vehicle, with a focus on reducing the concentration of drug required for treatment [64]. Interestingly, the component analysis of *A. sieberi* extract revealed the presence of several known antifungal components, including flavonoids (flavones, luteolin, apigenin), sesquiterpene lactones (Artemisin), cyclic sesquiterpens, bicyclic monoterpene glycosides, and sesquiterpene in plant extracts [65]. In this context, Galal et al. (2005) demonstrated potent antifungal activity of *Artemisinin* derivatives against *C. neoformans* compared to amphotericin B [66]. Similarly, apigenin displayed high antifungal activity against *T. mentagrophytes*, with an MIC value of 0.039 mg/mL [67]. Additionally, Ivanov et al. (2020) reported that luteolin inhibits the growth of C. albicans, with an MIC of 37.5 µg/mL [68]. The essential oil of *A. sieberi* displayed potent antifungal activity against *A. fumigatus*, with an MIC of 250 μg/mL [69], and C. glabrata, with MIC values ranging from 37.4 to 4781.3 μg/mL [70]. Our results are in agreement with those of Khatoon et al. (2019), who reported that AgNPs synthesized by *Artemisia annua* displayed potent antifungal effect against Candida species, with an MIC in the range of 80–120 μg/mL [71].

Several mechanistic pathways have been suggested to mediate the antifungal activity of AgNPs. The penetration of AgNPs through the cell membrane due to their nanosize could cause leakages in fungal membranes. Alternatively, AgNPs interact with oxidizers to generate silver ions, which in turn bind strongly with the proteins, enzymes, and DNA of fungi to break their bioprocesses, which consequently lead to fungal cell death [72]. In addition, AgNPs disturb the metabolism and proliferation of fungal cells [60]. In this study, the treatment of *A. fumigatus* with AS-AgNPs showed tortuous filaments as examined by SEM. This change in the wall feature might be a consequence of the perturbation cell membrane. In consistent, green-synthesized AgNPs caused structural changes in hyphae, including cell wall deformations, membrane disruption, and significant modifications in spore form and germination [73]. Similarly, [74] indicated that AgNPs might inhibit the growth of *A. flavus* by affecting cellular functions, which caused deformation in fungal hyphae, as examined by SEM in this study. Additionally, AgNPs could result in malformation and hypertrophy of fungal spores, which lead to the destruction and damage of spores. In this context, AgNPS result in breakage of hyphal tips, detached conidia, and damage to the surface of the fungal hyphae, which lead to the release of internal cellular materials, resulting in shrinkage of the hyphae [75]. Furthermore, AgNPs result in several modifications of fungal spores, as observed by TEM in this study. The main alterations were the change in the morphology and complete collapse of the spores after 36 h of exposure to biogenic AgNPs [76].

Understanding the infectious process of *A. fumigatus* is mainly based on the study of biofilm colonies rather than cells grown in planktonic form [77]. Several studies have reported the anti-biofilm activity of biosynthesized AgNPs by a decrease in their biomass and complex and formation of a vesicle transit station which play a major role in the hyphal morphogenesis [78,79]. In this context, we previously showed that biosynthesized AgNPs using different plant extracts display powerful antibiofilm action against *C. albicans* [25,26]. Similarly, [80] showed that AgNP-treated *C. albicans* biofilm have no true hyphae, and there was a clear reduction in the number of cells, and disruption of the cell wall was also observed. TEM analysis displayed that AgNPs not only attach and accumulate to the cell wall and membranes but also penetrate the cells and accumulate in the cytoplasm that might lead to rupturing of the cell wall and disintegration of the cytoplasmic membrane [56].

Our in vitro data showed no cytotoxicity of AgNPs up to a concentration of 100 ug/mL. In consistent, AgNPs showed no toxicity on human-adipose-derived stem cells [81] and the MCF-7 cell line [26]. However, the in vitro toxicity of AgNPs varies from one study to the other based on many factors, including manufacturing methods, size distributions, the cell line used, and culture conditions [82]. Almost all pre-clinical studies for the treatment of IPA in mice were based on using amphotericin B. These include a combination of amphotericin B and extract of cultured mushroom Lentinula edodes mycelia (AHCC^®^) [83], low-MWt amphotericin B–polymethacrylic acid nanoparticles [84], and poly(lactide-co-glycolode) (PLGA) nanoparticles encapsulating amphotericin B [85]. Thus, our study is the first to provide an alternative treatment for IPA using green-synthesized AgNPs from *A. sieberi* leaf extract.

Consistent with our results, in vivo infection of lungs with *A. fumigates* in IPA mice enhanced inflammatory-cytokine-mediated pathology by increasing the production of IL-17 [86], IL-1, and TNF by eosinophils and macrophages [87,88,89]. Interestingly, treatment of IPA mice with AS-AgNPs suppressed IPA-induced pro-inflammatory cytokines dramatically, most likely due to their direct effect on reducing the fungal burden by more than 50% and consequently inhibiting gliotoxin production by *A. fumigates*. Gliotoxin, an immune-suppressive mycotoxin, is one of the main virulence factors of *A. fumigatus* that is capable of damaging the epithelial/endothelial barriers of the respiratory tract [90,91]. Nanomaterials are shown to have interesting adsorption properties, which make them promising for mycotoxin elimination [92]. In this context, AgNPs are reported to exert an inhibitory effect on the production of some fungal toxins [93]. For example, AgNPs inhibit mycotoxin production (up to 80%) in *A. niger* and *P. chrysogenum* [94]. In addition, AgNPs decreased the mycotoxins produced by *Aspergillus* sp. by 81.1–95.5%. [95]. Thus, it is plausible that the therapeutic effect of AS-AgNPs in IPA is mediated by inhibiting the production of gliotoxin by *A. fumigatus.*

## 5. Conclusions

Invasive pulmonary aspergillosis (IPA) is a severe infection disease caused by *A. fumigatus*. In this study, we provided AgNPs biosynthesized with *A. sieberi* leaf extract (AS-AgNPs) as an alternative antifungal treatment for IPA in vivo. Our data demonstrated the inhibitory effect of AS-AgNPs on *A. fumigatus* growth and biofilm formation without exerting any cytotoxicity on human lung cells in vitro. Interestingly, the treatment of the IPA mouse model with AS-AgNPs demonstrated the therapeutic potential of AS-AgNPs in reducing lung tissue damage and suppressing the high levels of pro-inflammatory cytokines due to their direct effect on the fungal burden and gliotoxin production in the lungs.

## Data Availability

All materials are available from the corresponding author.

## Supplementary Materials

The following supporting information can be downloaded at: https://www.mdpi.com/article/10.3390/nano12010051/s1, Figure S1: Cytotoxicity effect of AS-AgNPs on primary mBMSCs; Figure S2: AS-AgNPs exert notoxicity in vivo in IPA mice; Table S1: In vitro antifungal activities of silver nanoparticles (AS-AgNPs) and amphotericin B (AMB) against *A. fumigatus*.
