# Supplementary material for: Effective Inhibition of Invasive Pulmonary Aspergillosis by Silver Nanoparticles Biosynthesized with Artemisia sieberi Leaf Extract
Source: Nanomaterials (Basel). 2021 Dec 25;12(1):51. doi: 10.3390/nano12010051 (PMC8746907; doi:10.3390/nano12010051)
Supplement: Supplementary file 1 [file nanomaterials-12-00051-s001.zip › nanomaterials-1510353-supplementary.pdf]

nanomaterials

Supplementary materials

Effective Inhibition of Invasive Pulmonary Aspergillosis by Silver Nanoparticles Biosynthesized with *Artemisia sieberi* Leaf Extract

Enas M. Ali [1,2] and Basem M. Abdallah [1,*]

[1] Department of Biological Sciences, College of Science, King Faisal University, Al-Ahsa 31982, Saudi Arabia; eabdelkader@kfu.edu.sa
[2] Department of Botany and Microbiology, Faculty of Science, Cairo University, Cairo, Egypt
* Correspondence: babdallallah@kfu.edu.sa; Tel.: +966-(013)-5899430

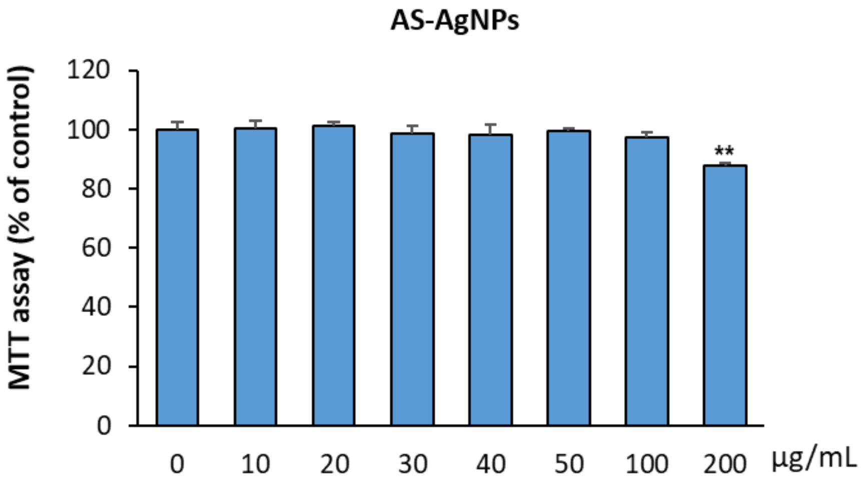

Figure S1. Cytotoxicity effect of AS-AgNPs on primary mBMSCs. The dose-dependent effect of AS-AgNPs on cell viability of cultured mBMSCs measured by MTT assay after 48 h of treatment . Values are mean ± SD of three independent experiments.

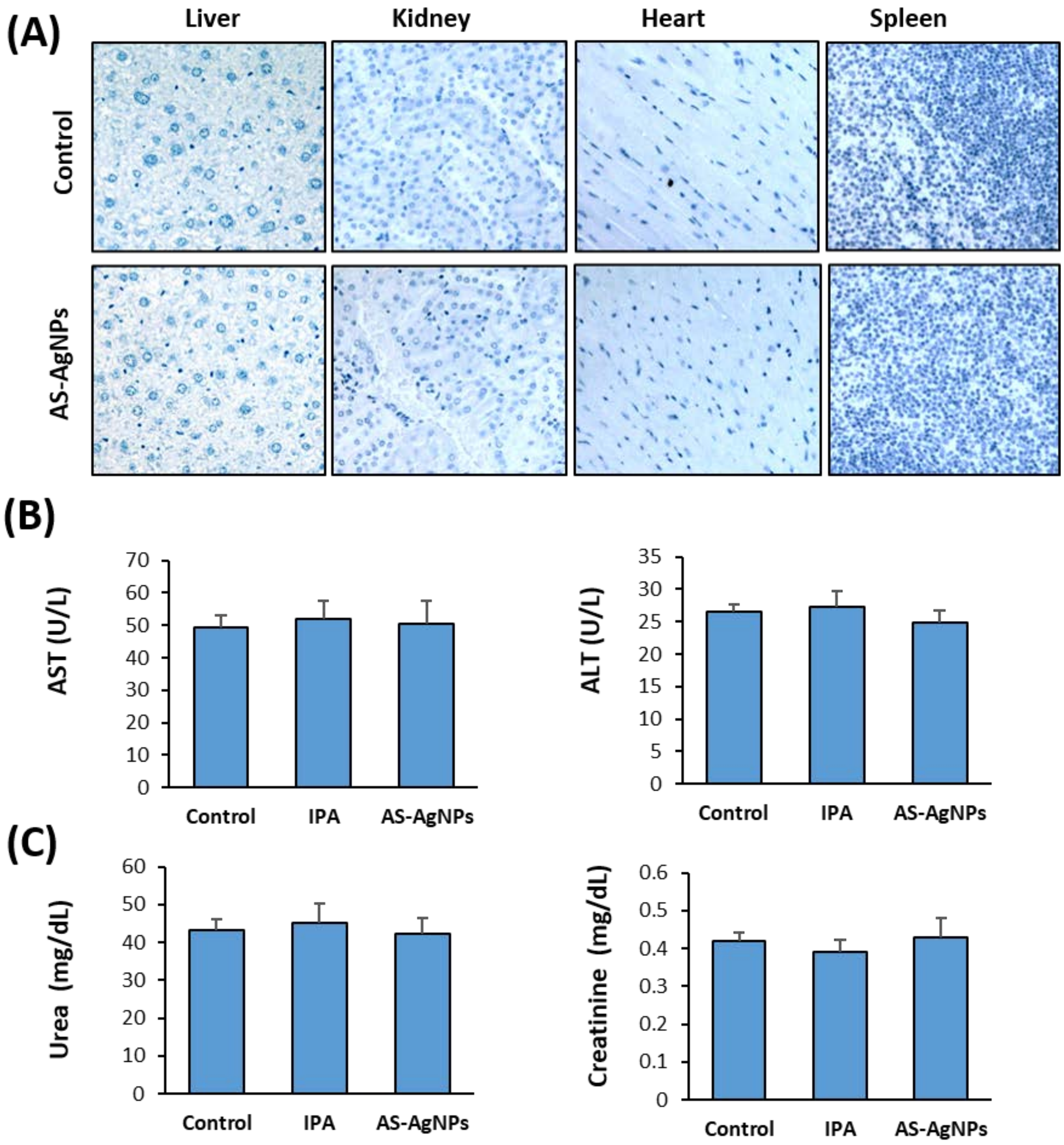

Figure S2. AS-AgNPs exert no toxicity in vivo in IPA mice. (**A**) Histological sections of liver, Kidney, heart and spleen with hematoxaline from IPA mice control and at 3 days post-AS-AgNPs instillation. Serum biochemical markers of liver function (**B**), including aspartate aminotransferase (AST) and alanine aminotransferase (ALT), and (**C**) renal function including urea and creatinine. Biochemical analysis were performed as described in M&M. Values are expressed as means ± SD (n=8 mice/group).

Table S1. In vitro antifungal activities of silver nanoparticles (AS-AgNPs) and amphotericin B (AMB) against *A. fumigatus*.

**Concentration gradient (μg/mL)**	**Antifungal agent**		
	**DMSO**	**AMB**	**AS- AgNPs**
	**IZD* (mm)**		
0	$_{a}0^{a}\pm0.0$	$_{a}0^{a}\pm0.0$	$_{a}0^{a}\pm0.0$
2	$_{a}0^{a}\pm0.0$	$_{b}10^{c}\pm0.7$	$_{b}7^{b}\pm0.7$
4	$_{a}0^{a}\pm0.0$	$_{c}14^{c}\pm0.5$	$_{c}10^{b}\pm0.5$
16	$_{a}0^{a}\pm0.0$	$_{d}17^{c}\pm0.5$	$_{d}13^{b}\pm0.8$
32	$_{a}0^{a}\pm0.0$	$_{e}27^{c}\pm0.5$	$_{e}17^{b}\pm0.8$
64	$_{a}0^{a}\pm0.0$	$_{f}35^{c}\pm0.5$	$_{f}23^{b}\pm0.8$
128	$_{a}0^{a}\pm0.0$	No growth	$_{g}30^{b}\pm0.8$
256	$_{a}0^{a}\pm0.0$		No growth
512	$_{a}0^{a}\pm0.0$		

Data are presented as mean ± SE (n=3 in each group).The means followed by same superscript letter in the same raw and those followed by the same subscript letter in the same column are not significantly different according to ANOVA and Duncan's multiple range tests at ($p < 0.05$).*Inhibition zone diameter.
